# Ketamine versus propofol for procedural sedation in pediatric pulsed dye laser therapy: a prospective randomized trial

**DOI:** 10.3389/fmed.2026.1824892

**Published:** 2026-04-30

**Authors:** Marija Stevic, Nina Ristic, Ivana Budic, Branislav Trifunovic, Vesna Marjanovic, Suzana Bojic, Dejan Zivorad Marković, Marija Jovanovski Srceva, Dragan Nenadic, Ana Vlajkovic Ivanovic, Lazar Milic, Dejan Nikolic

**Affiliations:** 1 University Children’s Hospital, Belgrade, Serbia; 2 Faculty of Medicine, University of Belgrade, Belgrade, Serbia; 3 Faculty of Medicine, University of Nis, Niš, Serbia; 4 University Clinical Center Nis, Nis, Serbia; 5 Saints Cyril and Methodius University in Skopje Medical Faculty, Skopje, North Macedonia; 6 Kent and Canterbury Hospital, Canterbury, United Kingdom

**Keywords:** ketamine, pediatric anesthesia, pediatric dermatology, port-wine stain, procedural sedation, propofol, pulsed dye laser

## Abstract

**Background:**

Pulsed dye laser (PDL) therapy is the gold standard for treating port-wine stains (PWS) in children but frequently requires procedural analgosedation due to pain and the need for immobility. The optimal sedative agent for repeated outpatient procedures remains unclear.

**Objective:**

To compare the safety and efficacy of propofol- versus ketamine-based analgosedation in children undergoing PDL therapy, with a primary focus on respiratory depression.

**Methods:**

In this prospective, randomized, controlled study, children aged 1–18 years (ASA I–II) were assigned to receive propofol or ketamine for PDL therapy. Standardized monitoring included electrocardiography, noninvasive blood pressure, pulse oximetry, and end-tidal CO₂. The primary outcome was respiratory depression, defined by predefined clinical and capnographic criteria. Secondary outcomes included hemodynamic instability, adverse events, drug consumption, procedural duration, sedation depth, and recovery time. Receiver operating characteristic (ROC) analysis was used to identify predictors of respiratory depression.

**Results:**

A total of 310 procedures were analyzed. Respiratory depression was significantly more frequent in the propofol group compared with the ketamine group (19.5% vs. 2.6%, *p* < 0.01). Ketamine was associated with higher rates of hypertension, tachycardia, psychomimetic effects, and postoperative nausea and vomiting (*p* < 0.01), whereas propofol was associated with more hypotension. Sedation and recovery times were longer with ketamine (*p* < 0.01), while sedation depth was comparable between groups. A procedural duration ≥13.5 min predicted respiratory depression in the propofol group (AUC 0.755).

**Conclusion:**

Both propofol and ketamine provide effective analgosedation for pediatric PDL therapy. Propofol is associated with a higher risk of respiratory depression, particularly during longer procedures, whereas ketamine offers greater respiratory stability but more other adverse effects. These findings support individualized anesthetic selection based on procedural duration and patient risk factors.

## 1 Introduction

Laser technology has become increasingly integrated into pediatric surgical and dermatologic practice over the past two decades. Advances in laser systems have expanded therapeutic indications to include vascular malformations, scars, keloids, tattoo removal, and various inflammatory and pigmentary skin disorders (1, 2).

Port-wine stain (PWS) is a congenital capillary malformation most commonly affecting the head and neck, with a reported prevalence of 0.3–0.9% in infants (2). Beyond cosmetic concerns, visible lesions in childhood may negatively affect psychosocial development and quality of life, prompting treatment often before school age (1, 2).

Pulsed dye laser (PDL) therapy represents the gold standard for PWS treatment; however, procedures can be painful and typically require multiple sessions. Older, cooperative children may tolerate local anesthesia with topical agents (EMLA 5%), but younger children generally require procedural analgosedation (PSA) (3). Commonly used agents for pediatric procedural sedation include benzodiazepines, opioids, propofol, and ketamine (4).

Ketamine is widely used in pediatric anesthesia due to its minimal respiratory depression, rapid recovery, bronchodilator properties, and maintenance of airway reflexes (5, 6). Its NMDA receptor antagonism mediates dissociative anesthesia and may have neuroprotective effects. Ketamine also produces sympathomimetic effects, increasing blood pressure and heart rate, which can be beneficial in hypotensive or shock states (6). Reported adverse effects include stridor, nystagmus, aspiration, laryngospasm, nausea, vomiting, nightmares, delirium, and agitation (5–7).

Propofol, an intravenous anesthetic commonly used for induction, maintenance, and procedural sedation, offers rapid onset and emergence, and decreases postoperative nausea and vomiting (8, 9). As a GABA-A agonist, propofol has a narrow therapeutic window in children and can induce dose-dependent respiratory depression, hypotension, bradycardia, and other complications (8, 10). Allergic reactions are rare but may occur in patients with known hypersensitivity.

Despite widespread use of both agents, evidence guiding anesthetic selection for pediatric laser therapy remains limited. Optimizing sedation strategies in pediatric outpatient procedures is increasingly important as minimally invasive interventions expand worldwide.

Unlike many other pediatric procedural sedation settings, pulsed dye laser (PDL) therapy presents several unique challenges. The procedure involves repetitive, high-intensity nociceptive stimuli delivered in short intervals, requiring sustained immobility despite its relatively brief duration. In addition, treatments are frequently performed on an outpatient basis and require multiple repeated sessions over time. These characteristics necessitate a careful balance between rapid onset, adequate analgosedation, cardiorespiratory stability, and fast recovery, thereby making the selection of an optimal sedative agent particularly important in this clinical setting.

The primary objective of this study was to compare the incidence of respiratory depression between propofol and ketamine based procedural analgosedation in children undergoing pulsed dye laser (PDL) therapy for port-wine stains.

Secondary objectives included comparison of hemodynamic stability, anesthetic-related adverse events, total drug consumption, procedural duration, depth of sedation, total sedation time, and recovery time.

## 2 Materials and methods

### 2.1 Study design and ethical approval

This prospective, randomized, controlled, open-label study was approved by the Ethics Committee of the University Children’s Hospital (approval number UCH-2019-25) and conducted in accordance with the principles of the Declaration of Helsinki. Written informed consent was obtained from the parents or legal guardians of all participants prior to enrollment.

### 2.2 Participants

**Figure 1:**
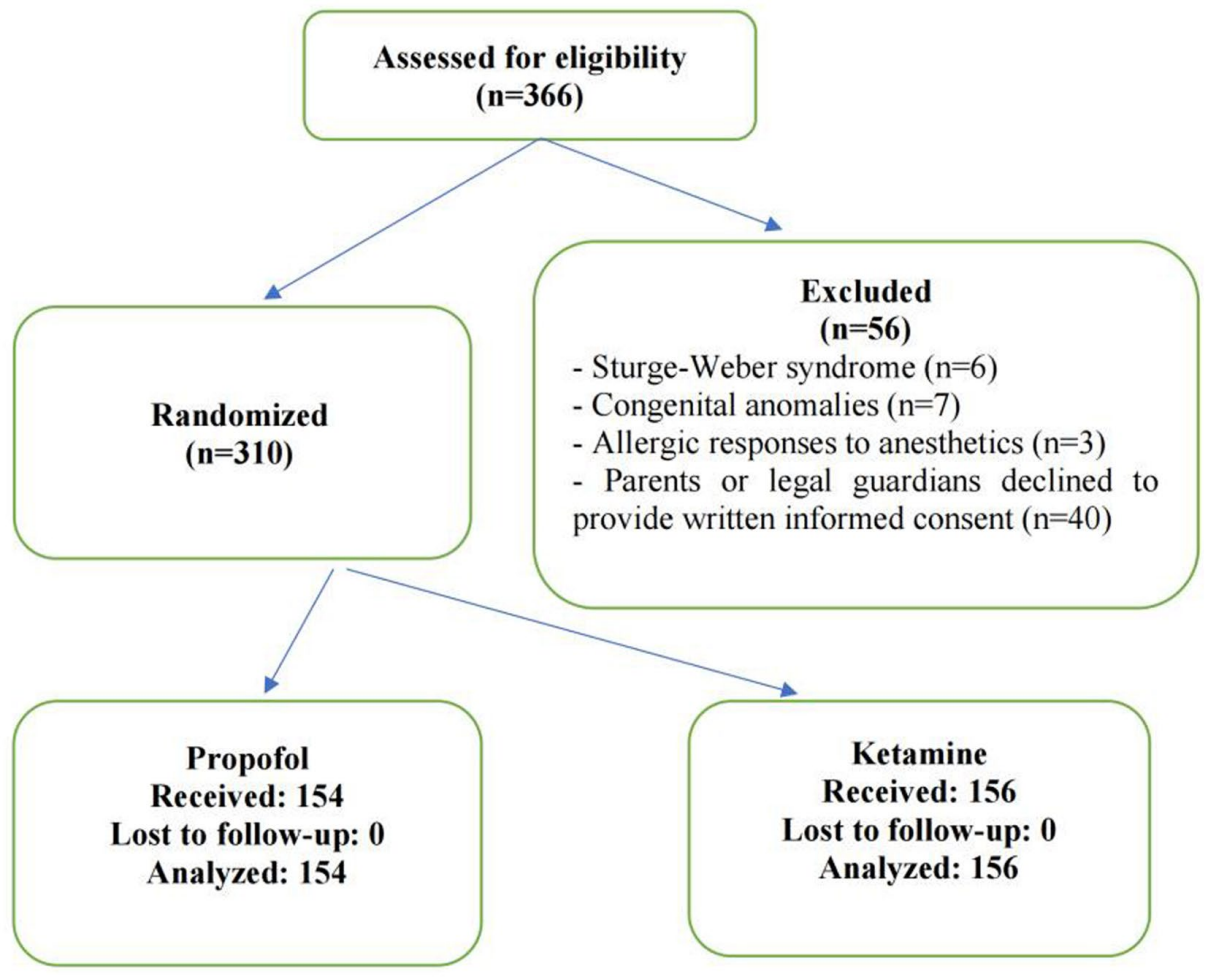
CONSORT flow diagram showing participant enrollment, exclusion, randomization, allocation, follow-up, and analysis in the propofol and ketamine groups.

Children aged 1–18 years with American Society of Anesthesiologists Physical Status (ASA-PS) class I–II undergoing pulsed dye laser (PDL) therapy for port-wine stains (PWS) between June 2021 and June 2024 were eligible for inclusion. Exclusion criteria included congenital disorders, Sturge–Weber syndrome, and known hypersensitivity to ketamine, propofol, or soy products. Participant flow through the study is presented in Figure 1, in accordance with CONSORT recommendations.

### 2.3 Randomization

Patients were randomly assigned in a 1:1 ratio to one of two sedation groups using a computer-generated randomization sequence.

Group I: Propofol (Propofol 1%, Fresenius Kabi Austria GmbH).

Group II: Ketamine (Ketamine hydrochloride USP, Rotexmedica).

Due to the nature of the interventions, the anesthesiologist administering the drugs was not blinded. However, surgeons and healthcare personnel involved in postoperative care and outcome assessment were blinded to group allocation.

### 2.4 Sedation protocol

All patients received oral midazolam syrup (0.5 mg/kg; maximum dose 10 mg) 30 min before the procedure as premedication. Procedural analgosedation was induced using one of the following regimens: propofol 1 mg/kg intravenously (propofol group) or ketamine 1 mg/kg intravenously (ketamine group). All patients additionally received fentanyl 1 μg/kg intravenously prior to initiation of laser therapy. Subsequent doses were titrated according to procedural requirements and clinical response.

### 2.5 Monitoring and perioperative management

Cardiorespiratory monitoring was initiated at the onset of sedation and included continuous electrocardiography (ECG), noninvasive blood pressure (BP), pulse oxygen saturation (SpO_2_), and nasal end-tidal carbon dioxide (ETCO_2_). Supplemental oxygen was administered via nasal cannula at a flow rate of 2 L/min using a Maquett Flow-i C20 anesthesia machine (Solna, Sweden). Following the procedure, patients remained under postoperative observation for 6 h in the presence of one parent or guardian.

### 2.6 Outcome measures

Baseline patient characteristics, including age, sex, and body weight, were recorded. Additional variables analyzed included the number of repeated procedures, complete laser treatment, total doses of administered anesthetic agents (propofol, ketamine, and fentanyl), procedure duration, depth of sedation, total sedation time, and recovery time. Adverse events recorded during the perioperative period included hypertension, tachycardia, hypotension, bradycardia, respiratory depression, laryngospasm, diplopia, nystagmus, hallucinations, and postoperative nausea and vomiting (PONV).

### 2.7 Definitions

ASA physical status classification was used to categorize patient health status (ASA I–VI) (11).

Respiratory depression during sedation was defined as the occurrence of any of the following events: increase in ETCO_2_ greater than 5 mmHg for ≥10 s; respiratory rate <8 breaths/min for ≥10 s; SpO_2_ < 90% for ≥10 s; apnea lasting >15 s; requirement for airway manipulation (jaw repositioning or bag-valve-mask ventilation) (12).

Hemodynamic stability was maintained through continuous monitoring and titration of anesthetic agents according to clinical response. In cases of hypotension (≤20% decrease from baseline), management included reduction of anesthetic dosing, intravenous fluid administration, and, if necessary, vasoactive support. Hypertension and tachycardia (≥20% increase from baseline), more commonly observed with ketamine, were managed by adjusting sedation depth and, when required, administering additional analgesia or sedative agents.

Continuous ECG monitoring was used throughout the procedure to detect rhythm disturbances and heart rate variability. Particular attention was paid in the ketamine group due to its known sympathomimetic effects, which may influence heart rate and blood pressure.

Sedation depth was carefully titrated to achieve a target modified Ramsay Sedation Score of 5, ensuring adequate immobility while minimizing the risk of cardiorespiratory instability (13).

Preventive measures for postoperative nausea and vomiting (PONV) included minimizing opioid use where possible and ensuring adequate hydration. PONV was treated with ondansetron (0.1 mg/kg intravenously) when clinically indicated.

Pain management was achieved using a multimodal approach, including additional doses of fentanyl that were administered based on clinical signs of inadequate analgesia during the intervention, and topical anesthesia (EMLA cream) after laser treatment.

Duration of anesthesia (procedural sedation) was defined as the time from induction of anesthesia to spontaneous eye opening.

Total sedation time was defined as the interval from administration of sedative drugs to spontaneous eye opening.

Duration of laser intervention was defined as the time from the first laser pulse to the completion of the procedure.

Postoperative nausea and vomiting (PONV) was assessed using a Numeric Rating Scale (NRS) ranging from 0 to 10, where 0 indicates no symptoms and 10 indicates severe symptoms.

Recovery time was defined as the time required to achieve a Steward Recovery Score (SRS) greater than 6 (14).

Psychomimetic effects were defined as the occurrence of agitation, hallucinations, delirium, or abnormal perceptual experiences during recovery (5).

### 2.8 Laser safety

All laser procedures were performed in the operating room. Protective eyewear was worn by the patient, surgeon, and anesthesiologist during the procedure. Operating room windows were covered with protective foil to ensure laser safety.

### 2.9 Statistical analysis

Continuous variables are presented as mean ± standard deviation (SD) or median (interquartile range, IQR), depending on data distribution. Normality of distribution was assessed prior to analysis. Comparisons between groups were performed using Student’s *t*-test for normally distributed variables and the Mann–Whitney U test for non-normally distributed variables. Categorical variables are presented as frequencies and percentages and were compared using the *χ*^2^ test or Fisher’s exact test as appropriate. Receiver operating characteristic (ROC) curve analysis was used to determine the optimal cut-off value for predicting respiratory depression using anesthesia duration. The area under the curve (AUC) with 95% confidence intervals was calculated. Statistical analyses were performed using SPSS software version 17.0 (SPSS Inc., Chicago, IL, USA). A two-sided *p*-value <0.05 was considered statistically significant. *Post hoc* power analysis confirmed that the study was adequately powered to detect clinically significant differences in the primary outcome.

### 2.10 Sample size calculation

*A priori* sample size estimation was performed based on the primary outcome, respiratory depression. Based on available literature and institutional experience, a clinically relevant difference of approximately 15% between groups was assumed. Using a two-sided significance level of 0.05 and 80% power, the required sample size was calculated as 152 participants per group. To ensure adequate statistical power and account for potential exclusions, approximately 155 patients were enrolled in each study arm.

## 3 Results

### 3.1 Participant characteristics

The mean age of participants was 4.6 ± 2.9 years in the propofol group and 4.8 ± 3.5 in the ketamine group, with no significant differences between the groups regarding age, sex, or body weight. The number of repeated laser procedures was significantly higher in the propofol group (median 4) compared with the ketamine group (median 3; Mann–Whitney *U* test, *p* < 0.01) (Table 1).

**Table 1:** Patient demographics and procedural characteristics.

Variable	Propofol (*n* = 154)	Ketamine (*n* = 156)	Test	*p*
Age (years), mean ± SD	4.6 ± 2.9	4.8 ± 3.5	*T*-test	0.658
Gender (m/f)	67/87	78/78	*χ*^2^ test	0.258
Body weight (kg)	20.9 ± 10.4	22.3 ± 12.8	*T*-test	0.307
Repeated procedures, median (range)	4 (1–12)	3 (1–8)	Mann–Whitney *U*	0.000
Completed procedures, *n* (%)	72 (46.8%)	30 (19.2%)	*χ*^2^ test	0.000

SD—standard deviation.

### 3.2 Procedural completion and drug consumption

Complete treatment was achieved in 72 patients (46.8%) in the propofol group and 30 patients (19.2%) in the ketamine group (*χ*^2^ = 26.59, *p* < 0.01) (Table 1). Total fentanyl consumption was significantly higher in the propofol group compared with the ketamine group (*t* = 4.09, df = 308, *p* < 0.01).

### 3.3 Cardiorespiratory outcomes

Significant differences in hemodynamic responses were observed between the groups.

Hypertension occurred more frequently in the ketamine group compared with the propofol group (17.9% vs. 5.8%, *p* < 0.01), as did tachycardia (21.8% vs. 4.5%, *p* < 0.01). Respiratory depression occurred in 19.5% of patients in the propofol group compared with 2.6% in the ketamine group. No cases of bradycardia were recorded in either group.

### 3.4 Other adverse events

Adverse effects including diplopia, nystagmus, hallucinations, and postoperative nausea and vomiting (PONV) occurred more frequently in the ketamine group (*p* < 0.01) (Table 2).

**Table 2:** Cardiorespiratory and other adverse events.

Adverse event	Propofol (*n* = 154)	Ketamine (*n* = 156)	Test	*p*
Hypertension	9 (5.8%)	28 (17.9%)	*χ*^2^ test	0.000
Tachycardia	7 (4.5%)	34 (21.8%)	*χ*^2^ test	0.000
Hypotension	30 (19.5%)	0 (0%)	Fisher’s exact	0.000
Respiratory depression	30 (19.5%)	4 (2.6%)	Fisher’s exact	0.000
Laryngospasm	2 (1.4%)	1 (1.54%)	Fisher’s exact	0.684
Diplopia	0 (0%)	48 (30.8%)	Fisher’s exact	0.000
Nystagmus	0 (0%)	75 (48.1%)	Fisher’s exact	0.000
Hallucinations	1 (0.6%)	41 (26.3%)	Fisher’s exact	0.000
Postoperative nausea and vomiting (PONV)	1.32 (1–3)	1 (1–2)	Mann–Whitney *U*	0.000
Bradycardia	0 (0%)	0 (0%)	–	NS

NS—not significant.

### 3.5 Sedation and recovery characteristics

Depth of sedation was comparable between the groups, with a median Ramsay sedation score of 5 (*p* > 0.05). However, total sedation time and recovery time were significantly longer in the ketamine group (*p* < 0.01; Table 3).

**Table 3:** Sedation depth, duration and recovery characteristics.

Characteristics	Propofol (*n* = 154)	Ketamine (*n* = 156)	Test	*p*
Duration of laser intervention (min), mean ± SD	7.51 ± 3.99	7.18 ± 3.95	Mann–Whitney *U*	0.327
Ramsay sedation Scale (1–6), Ramsay sedation score, median	5 (4–6)	5 (4–6)	Mann–Whitney *U*	0.366
Total sedation time (min), mean ± SD	14.17 ± 5.46	16.51 ± 4.19	*T*-test	0.000
Recovery time (min), mean ± SD	9.08 ± 1.12	11.31 ± 2.35	*T*-test	0.000

SD—standard deviation.

### 3.6 Predictors of respiratory depression

**Figure 2:**
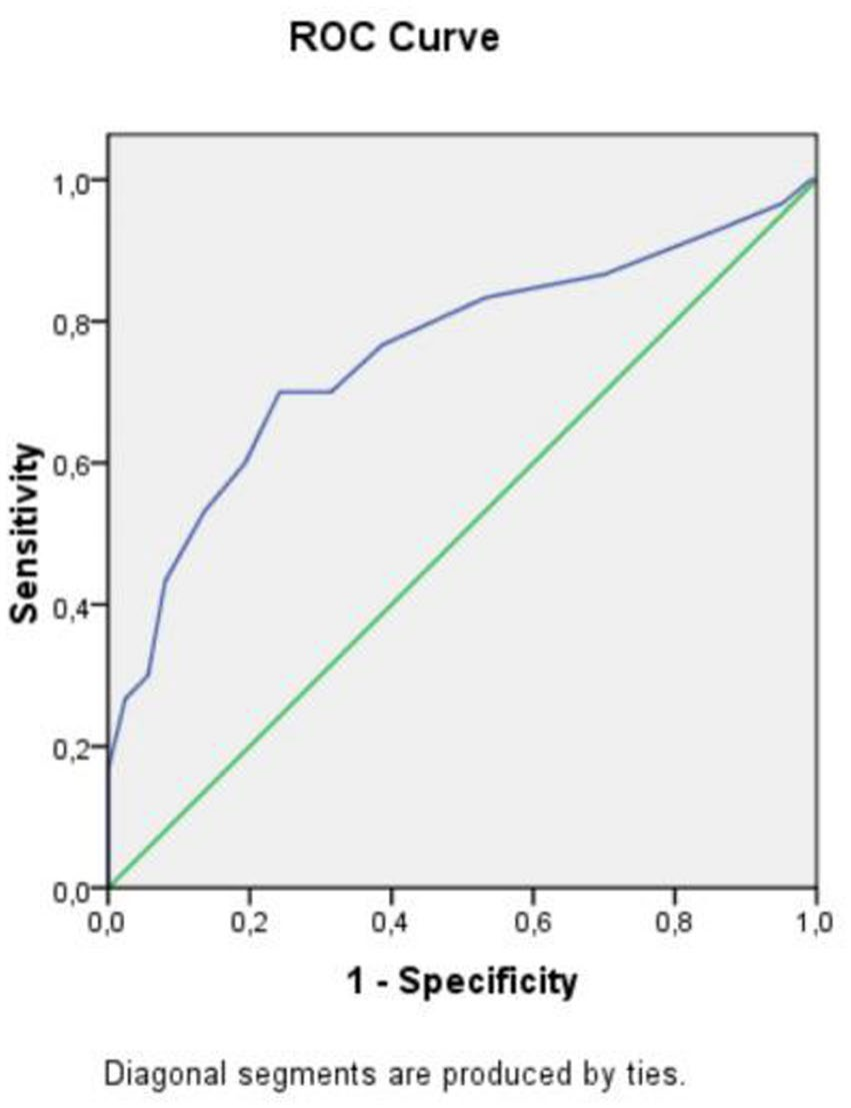
ROC curve analysis.

Receiver operating characteristic (ROC) analysis demonstrated that anesthesia duration ≥13.5 min in the propofol group was associated with a significantly increased risk of respiratory depression (AUC 0.755; 95% CI 0.658–0.851, *p* < 0.01), with a sensitivity of 70% and specificity of 75.8% (Figure 2).

## 4 Discussion

Laser therapy has become an important modality for the treatment of various skin disorders, enabled by advances in laser technology that allow minimally invasive interventions (15, 16). However, pulsed dye laser (PDL) therapy in children poses distinct anesthetic challenges, including repetitive painful stimuli, the requirement for immobility, and the frequent repetition of procedures, which demand meticulous selection of sedation strategies.

In the present study, no statistically significant differences were observed between the groups regarding gender distribution, body weight, or age. The mean age of patients in both groups was approximately 4 years, which is consistent with previous studies recommending pulsed dye laser (PDL) therapy in children younger than 10 years (17). In our study, the average number of PDL procedures per patient was four in the propofol group and three in the ketamine group, while complete healing occurred in 46.8% of patients in the propofol group and 19.2% in the ketamine group. The number of repeated procedures and completion rates in our study may be influenced by individual treatment response, lesion characteristics (e.g., size, location, and depth), and clinical decision-making during follow-up, rather than sedation type alone. Additionally, variability in scheduling, patient adherence, and physician preference may have contributed to this difference.

The principal finding of this study is that respiratory depression occurred significantly more frequently in the propofol group compared with the ketamine group, confirming our primary hypothesis. This finding is consistent with previous studies demonstrating a higher incidence of respiratory adverse events associated with propofol-based sedation in pediatric populations (18–22). The pharmacological profiles of the two agents explain the observed difference. Propofol produces dose-dependent respiratory depression by central inhibition of respiratory drive, whereas ketamine preserves spontaneous respiration and airway reflexes through its dissociative mechanism of action (5, 6).

Importantly, our study extends the literature by identifying a clinically relevant procedural duration threshold (≥13.5 min) associated with an increased risk of respiratory depression. While prior studies have reported risk factors for respiratory events, few have provided a practical, time-based parameter for procedural planning. This finding may therefore have direct clinical utility in risk stratification. With respect to secondary outcomes, hemodynamic responses differed significantly between the groups, with hypertension and tachycardia more frequently observed in the ketamine group and hypotension more common in the propofol group. These findings are in agreement with previous reports (20, 21). From a pharmacological perspective, we can anticipate these differences. Ketamine’s sympathomimetic effects lead to increased heart rate and blood pressure, whereas propofol is associated with vasodilation and myocardial depression, resulting in hypotension. Compared with some previous studies reporting higher rates of hypotension with propofol, the incidence observed in our cohort was relatively moderate. This discrepancy may be explained by the use of structured perioperative management protocols and careful titration of sedation depth, which likely contributed to improved hemodynamic control in our study.

We found that total sedation time and recovery time were significantly longer in the ketamine group, despite comparable sedation depth between groups. These findings are consistent with earlier studies (18, 19) and reflect the longer elimination profile and psychomimetic recovery phenomena associated with ketamine. Conversely, the abbreviated recovery profile associated with propofol is consistent with its rapid redistribution and elimination. However, this benefit must be weighed against its less favorable respiratory safety profile, especially during extended procedures. Our results demonstrate that psychomimetic adverse effects and PONV were more frequent in the ketamine group, whereas respiratory complications predominated in the propofol group, consistent with previous literature (18, 21). Additionally, total fentanyl consumption was higher in the propofol group, which can be explained by the lack of intrinsic analgesic properties of propofol. In contrast, ketamine provides both sedation and analgesia through NMDA receptor antagonism, reducing the need for supplemental opioids. This finding aligns with current recommendations supporting ketamine as part of multimodal analgesia strategies in pediatric anesthesia (23).

Patients in the propofol group received higher cumulative doses of anesthetic agents, raising the question of potential pharmacological tolerance during repeated procedures. Existing evidence on propofol tolerance in children remains inconclusive, with some studies suggesting minimal tolerance development, while others report variable responses (9, 24, 25). In the context of PDL therapy, where repeated short procedures are common, this aspect warrants further investigation. Variability in procedural frequency, dosing strategies, and patient populations may account for the differences between our findings and previous reports. In general, our results are consistent with existing knowledge regarding the safety of propofol and ketamine in pediatric procedural sedation. However, this study adds novel evidence by focusing specifically on the PDL setting, which differs from other procedural contexts due to repetitive, short-duration interventions; the requirement for immobility despite brief procedures; and cumulative exposure to sedation over time. These factors may influence both drug selection and risk profiles, potentially explaining differences in effect magnitude compared with studies conducted in emergency or diagnostic settings.

### 4.1 Clinical implications

From a clinical perspective, both propofol and ketamine provide effective sedation for short PDL procedures. However, propofol offers faster recovery but carries a higher risk of respiratory depression, and ketamine ensures greater respiratory stability but is associated with hemodynamic stimulation and psychomimetic effects. The identification of a 13.5-min procedural threshold is particularly relevant, suggesting that longer procedures may benefit from enhanced airway control strategies or alternative anesthetic approaches.

### 4.2 Future directions

Given the increasing use of repeated procedural sedation in children, further multicenter studies are needed to evaluate long-term neurodevelopmental outcomes, cumulative effects of repeated sedation exposure, and optimization of drug combinations and dosing strategies.

### 4.3 Limitations

This study has several limitations. First, it was conducted at a single tertiary pediatric center, which may limit the generalizability of the findings. Second, blinding of the anesthesiologist was not feasible due to the nature of drug administration, which may introduce potential performance bias. Third, only ASA I–II patients were included; therefore, the results may not be directly applicable to children with significant comorbidities. Fourth, repeated procedures in some patients may introduce intra-individual variability, although each sedation event was analyzed independently.

Finally, the study focused on short-term perioperative outcomes, and long-term neurodevelopmental and behavioral effects of repeated sedation exposure were not assessed. Although an *a priori* sample size calculation was performed, assumptions regarding the expected incidence of respiratory depression were based on available literature and institutional experience, which may limit precision.

## 5 Conclusion

Laser therapy for port-wine stain represents an expanding field within pediatric dermatologic and surgical practice, where safe and effective anesthetic management is essential. In this prospective randomized trial, both ketamine and propofol provided adequate sedation for short pulsed dye laser procedures in children. Ketamine was associated with greater hemodynamic stimulation and psychomimetic effects, whereas propofol demonstrated a higher incidence of respiratory depression, particularly when the duration of the procedure exceeded 13.5 min. These findings highlight the importance of individualized anesthetic planning based on expected procedure duration and patient-specific risk factors to optimize perioperative safety.

## Data Availability

The raw data supporting the conclusions of this article will be made available by the authors, without undue reservation.
